# Resveratrol Pretreatment Attenuates Concanavalin A-induced Hepatitis through Reverse of Aberration in the Immune Response and Regenerative Capacity in Aged Mice

**DOI:** 10.1038/s41598-017-02881-z

**Published:** 2017-06-02

**Authors:** Tse-Hung Huang, Chin-Chang Chen, Hsuan-Miao Liu, Tzung-Yan Lee, Sue-Heui Shieh

**Affiliations:** 1 Department of Traditional Chinese Medicine, Chang Gung Memorial Hospital, Keelung, Taiwan, ROC; 2 School of Traditional Chinese Medicine, Chang Gung University, Taoyuan, Taiwan, ROC; 3 School of Nursing, National Taipei University of Nursing and Health Sciences, Taipei, Taiwan, ROC; 4 Graduate Institute of Traditional Chinese Medicine, Chang Gung University, Taoyuan, Taiwan, ROC; 5 Division of Urology, Department of Surgery, Chang Gung Memorial Hospital, Linkou, Taoyuan, Taiwan, ROC; 6 Institute of Pharmacology, National Yang-Ming University, Taipei, Taiwan, ROC; 7 Department of Nursing, Chang Gung University of Science and Technology, Taoyuan, Taiwan, ROC

## Abstract

Loss of regenerative capacity plays a critical role in age-related autoimmune hepatitis. Evidence implicates SIRT1 and p66^shc^ in cell senescence, apoptosis, oxidative stress, and proliferation. This study investigated the effect of resveratrol on concanavalin A (Con A)-induced hepatitis in aged mice and the roles of SIRT1 and p66^shc^. Aged mice were administrated resveratrol (30 mg/kg orally) seven times at an interval of 12 h before a single intravenous injection of Con A (20 mg/kg). Results showed that the cytokines, TNF-α, IL-6, IFN-γ, and MCP-1, as well as infiltration of macrophages, neutrophils, and T lymphocytes in liver were dramatically enhanced in the mice given only Con A. The aged mouse livers showed markedly raised oxidative stress and cell apoptosis. This oxidative stress further aggravated regenerative dysfunction as indicated by the decreased levels of Ki67, PCNA, Cyclin D1, and Cdk2. Conversely, these phenomena were attenuated by pretreatment with resveratrol. Moreover, resveratrol suppressed the elevation of p66^shc^ in the liver by reversing Con-A-mediated downregulation of SIRT1. The findings suggest that resveratrol protected against Con A-induced hepatitis in aged mice by attenuating an aberration of immune response and liver regeneration, partially *via* the mechanism of SIRT1-mediated repression of p66^shc^ expression.

## Introduction

The aging of the majority of the world’s population is a recent phenomenon that has emerged as a direct consequence of the rise in human life expectancy. Aging is considered a risk factor for chronic diseases, including autoimmune disorders^1, 2^. A large body of evidence revealed that age-associated disturbances in innate immunity (immune response of neutrophils and macrophages) and adaptive immunity (B cell and T cell development) are higher than in young individuals^3–5^. Autoimmune hepatitis is an idiopathic inflammatory disease of the liver that is characterized by a loss of self-tolerance, leading to the appearance of autoantibodies and dense lymphoplasmacytic inflammatory infiltrates in the portal tracts, with piecemeal necrosis of periportal hepatocytes^6^. Previous studies suggest that 20% of patients develop autoimmune hepatitis after 60 years of age, and the disease is frequently progressive and unexpected because ascites and cirrhosis are common manifestations at presentation, with few other symptoms^7–9^. Although most elderly patients respond well to corticosteroid therapy^8^, variant or overlapping syndromes are worthy of consideration when unexpected disease features arise.

The loss of regenerative capacity is the most dramatic age-associated alteration in liver tissue after surgical resection or chemical injury^10, 11^. Enkhbold *et al*. indicate that impaired liver regeneration in mice is due to aging, which is expressed by an increase of autophagy and apoptosis after partial hepatectomy^10^. In addition, the reduced response to oxidative stress and decreased expression of growth-regulatory factors also contribute to reduced hepatic regenerative capacity and further increase susceptibility to certain liver diseases among the aged^12^. Moreover, the activated immune cells restrain regenerating liver cells. T cell-mediated murine hepatitis, induced by concanavalin A (Con A), could enhance transforming growth factor-β (TGF-β) and interferon-γ (IFN-γ) expression, reduce hepatocellular regeneration, and induce natural killer (NK) cell-sensitive oval cells following partial hepatectomy^13^. Notably, Zhang *et al*. suggest that interleukin-22 (IL-22) plays a protective role in the survival of mice with Con A-induced hepatitis after partial hepatectomy by enhancing liver regeneration^14^. Furthermore, augmented liver regeneration represses Con A-induced hepatitis in mice via nuclear factor-κB (NF-κB) inhibition of T cells^15^.

Sirtuins are a mammalian family of NAD^+^-dependent histone deacetylases. SIRT1 regulates cell apoptosis, senescence, metabolism, and proliferation, and thereby influences multiple biological conditions including longevity, obesity, age-related diseases, and cancer^16, 17^. Recent studies show that SIRT1 null mice developed an autoimmune-like disease accompanied by accumulation of immune complexes in the liver and kidney^18^. P66^shc^, an isoform of ShcA adaptors, plays a role in regulating cellular responses to oxidative stress, apoptosis, and aging^19, 20^ whereas p66^shc^ knockout mice are resistant to oxidative stress and age-related pathologies^21–23^. Additionally, p66^shc^ is negatively regulated by SIRT1 in accordance with evidence indicating that SIRT1 inhibited p66^shc^ expression in diabetic mice and protected against hyperglycemia-induced endothelial dysfunction and senescence^24, 25^. Xu *et al*. suggests that the protective effects of salvianolic acid A on Con A-induced hepatitis in mice are correlated with SIRT1-mediated repression of the p66^shc^ pathway^26^. Moreover, p66^shc^ has a crucial function in the impairment of liver regeneration in aged mice by triggering oxidative stress and apoptosis^27^. However, it is still unknown whether SIRT1 and p66^shc^ are involved in the immune dysregulation and dysfunction of liver regeneration in elderly humans with autoimmune hepatitis.

Resveratrol, a phenolic compound that is found in various plants, including red grapes and their derivatives^28^, has many health advantages. These benefits include anti-inflammatory and antioxidant properties that enhance the production of antioxidant enzymes^29^. It is reported that resveratrol provides protection against ongoing liver damage resulting from hepatotoxins such as acetaminophen, ethanol, and carbon tetrachloride^30–32^. Zhou *et al*. revealed that resveratrol protects against Con A-induced autoimmune hepatitis by decreasing cytokine expression in mice through modulation of the hedgehog signal pathway^33^. Therefore, we investigated whether pretreatment with resveratrol could prevent Con A-induced hepatitis in aged mice through reversal of immune system and liver regeneration impairment. Additionally, we sought to determine the roles of SIRT1 and p66^shc^ involved in Con A-induced autoimmune hepatitis in aged mice.

## Results

### Effect of resveratrol on Con A-induced hepatitis in aged mice

**Figure 1:**
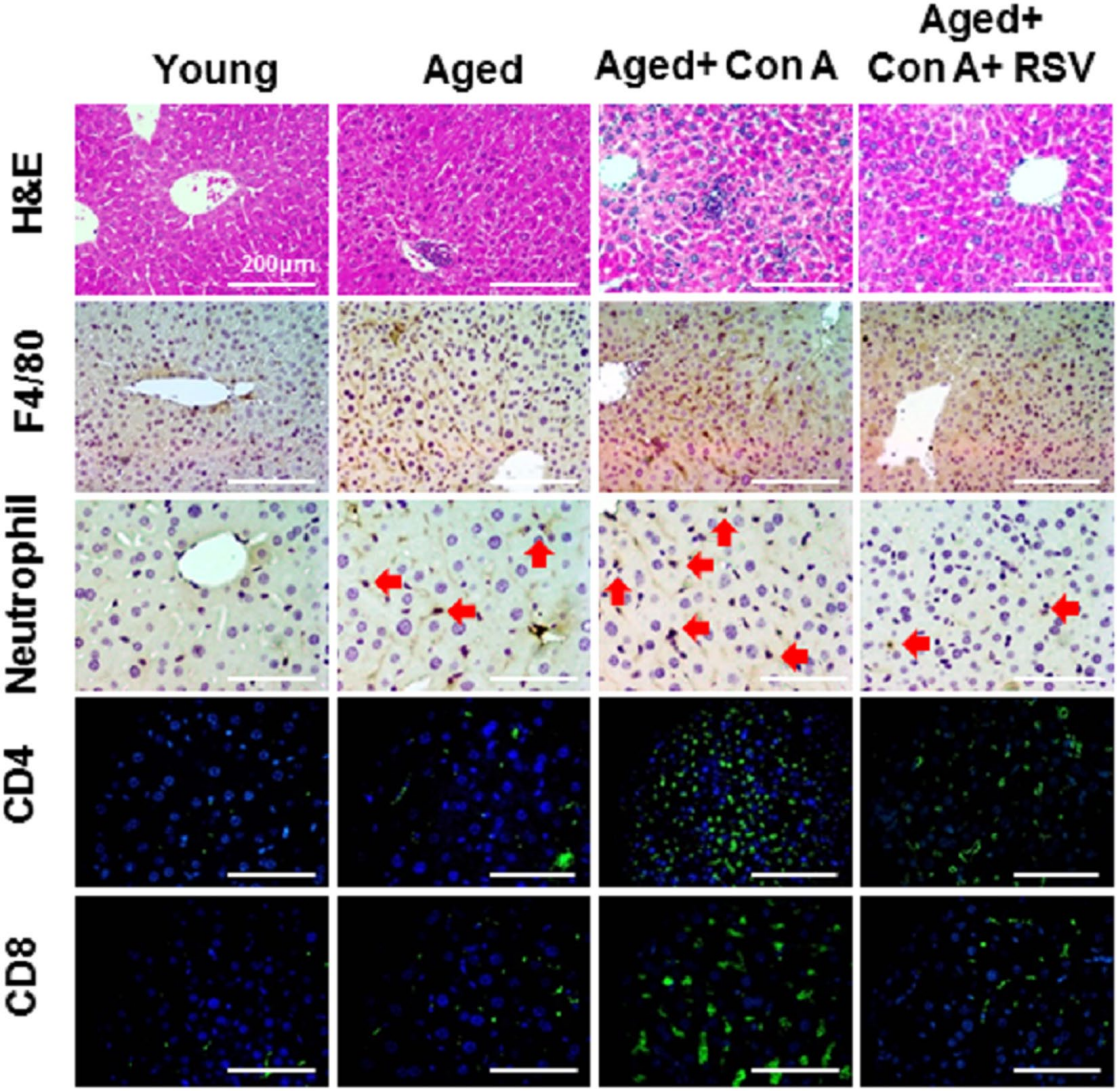
Resveratrol ameliorated Con A-induced histopathological change in the liver tissue of aged mice. H&E, IHC, and IF staining by group show infiltration of macrophages and neutrophils in liver (dark brown in color with red arrows indicating neutrophils; F4/80 and neutrophil stain, respectively). The T helper and T cytotoxic lymphocytes were stained with CD4 and CD8 antibodies, respectively. DAPI is the nuclear stain. All results are shown at 200 × magnification and the length of scale bar was indicated as 200 μm.

As shown in Supplementary Fig. 1, the small and scattered necro-inflammatory foci with polymorphonuclear cell infiltration were observed in liver portal area of young mice that were challenged with Con A. In contrast, a massive infiltration of T lymphocytes and large areas of necrosis were observed in the liver lobules of aged mice that were challenged with Con A. Furthermore, immunohistochemical staining showed there was a markedly increase in neutrophils and macrophages (see F4/80 stain) recruited to the liver post-Con A treatment in aged mice, notwithstanding less took place in young group. Western blotting data showed that hepatic levels of PAI-1, MCP-1 and NF-κB p65 in aged mice were higher than young group (Supplementary Fig. 2). Meanwhile, Con A treatment markedly increased the inflammatory factors expression, including PAI-1, iNOs, MCP-1 and NF-κB p65 in both young and aged mice, particularly in aged mice (Supplementary Fig. 2). Since the goal of the current work was trying to determine if resveratrol pretreatment can attenuates Con A-induced inflammatory response. As indicated in Table 1, aged mice showed significantly increased plasma levels of alanine transaminase (ALT), aspartate transaminase (AST), tumor necrosis factor-α (TNF-α), IL-6, and interferon-γ (IFN-γ) but not monocyte chemoattractant protein-1 (MCP-1) compared with young mice. Additionally, these plasma inflammatory cytokines dramatically increased in aged mice after Con A injection. Figure 1 shows the characteristic features of liver pathology such as few inflammatory cells in the portal area and large hepatocytes with pink-staining cytoplasm and round nuclei. The features of advanced liver injury include areas of scattered inflammatory and necrotic foci with polymorphonuclear cell infiltration, massive infiltration of T lymphocytes, and large areas of necrosis in the liver lobules in aged mice exposed to Con A (H&E, CD4- and CD8-IF stains). Con A treatment stimulated infiltration of macrophages and neutrophils in the mice (F4/80 and neutrophil stains). In contrast, resveratrol pretreatment significantly attenuated Con A-induced plasma inflammatory cytokines and liver necrosis in aged mice.

**Table 1:** Effect of RSV on plasma inflammatory cytokines in aged mice treated with Con A.

	Young	Aged	Aged + Con A	Aged + Con A + RSV
ALT (IU/L)	110.21 ± 20.43 ^a^	250.48 ± 28.55^b^	853.13 ± 40.98^c^	351.98 ± 34.85^d^
AST (IU/L)	90.56 ± 24.89^a^	180.96 ± 20.44^b^	753.98 ± 42.56^c^	295.86 ± 27.85^d^
TNF-α (pg/mL)	53.24 ± 9.26^a^	129.13 ± 20.55^b^	314.98 ± 45.98^c^	159.73 ± 22.98^d^
IL-6 (pg/mL)	49.53 ± 19.83^a^	110.23 ± 18.51^b^	513.47 ± 38.98^c^	215.87 ± 24.56^d^
IFN-γ (pg/mL)	31.73 ± 11.24^a^	66.76 ± 15.59^b^	285.95 ± 25.13^c^	137.75 ± 22.85^d^
MCP-1 (pg/mL)	107.85 ± 20.52^a^	135.45 ± 25.95^a^	837.98 ± 39.53^b^	203.13 ± 25.33^c^

Data are expressed as means ± S.E.M. and each group consists of 7-8 mice. Values marked by different letters denote statistically significant differences (p < 0.05) between groups according to Tukey’s post hoc test. ALT, alanine transaminase; AST, aspartate transaminase; TNF-α, tumor necrosis factor-α; IL-6, interleukin-6; IFN-γ, interferon-γ; MCP-1, monocyte chemoattractant protein-1.

**Figure 2:**
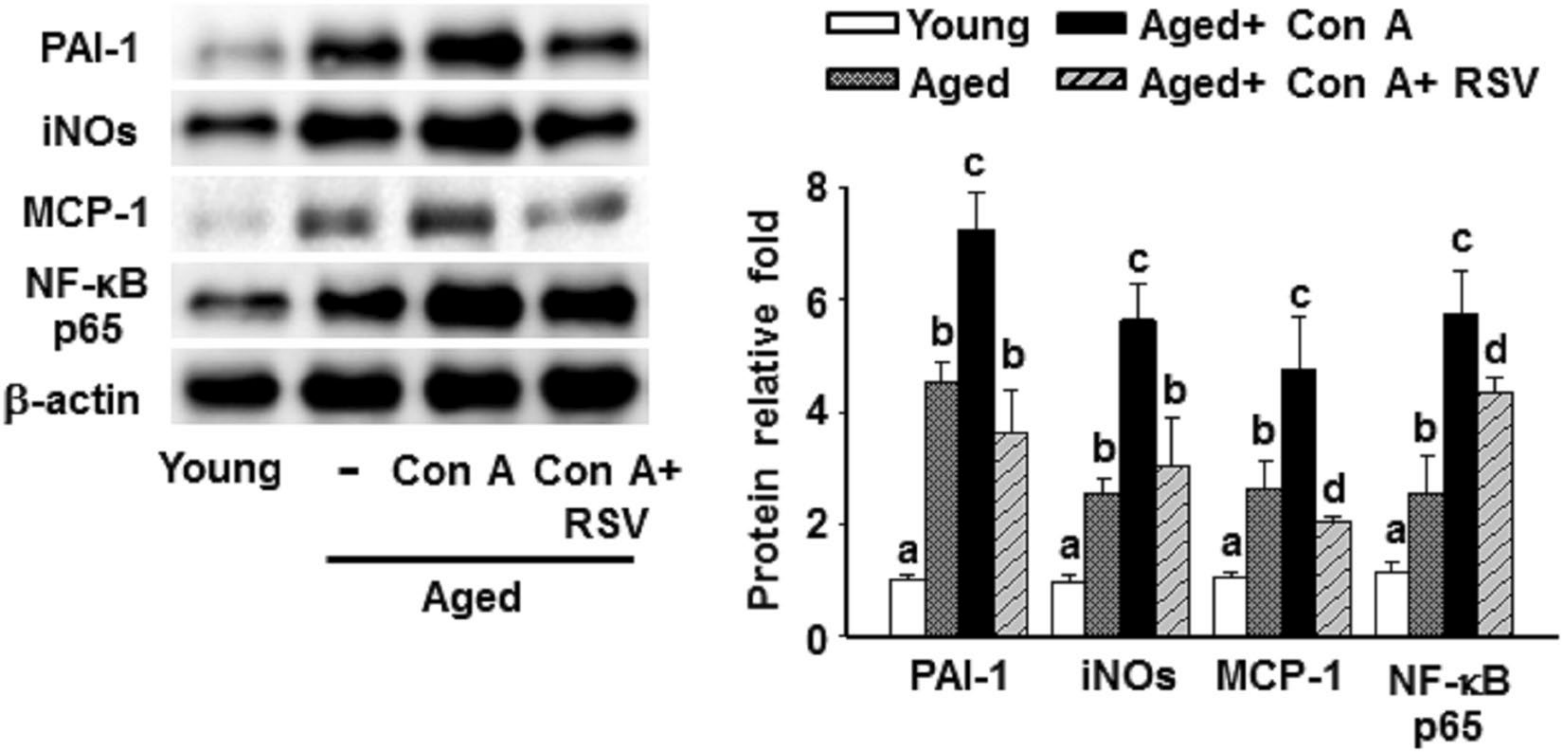
Resveratrol reduced Con A-induced inflammatory responses in the liver tissues of aged mice. Hepatic protein levels of PAI-1, iNOs, MCP-1, and NF-κB p65 were detected by Western blotting. The results were quantified by densitometry and represented as the means ± SEMs from three independent measurements. Values marked by different letters denote statistically significant differences (p < 0.05) between groups, according to Tukey’s post hoc test.

In addition, Western blotting analyses showed that hepatic levels of plasminogen activator inhibitor-1 (PAI-1), inducible nitric oxide synthase (iNOs), MCP-1, and NF-κB p65 in aged mice were higher than in the young group of mice (Fig. 2). Con A treatment dramatically increased expression of these inflammatory factors in the livers of aged mice. Resveratrol treatment significantly reduced these effects in aged mice with Con A- induced hepatitis (Fig. 2).

### Effects of resveratrol on SIRT1 and p66^shc^ in aged mice treated with Con A

**Figure 3:**
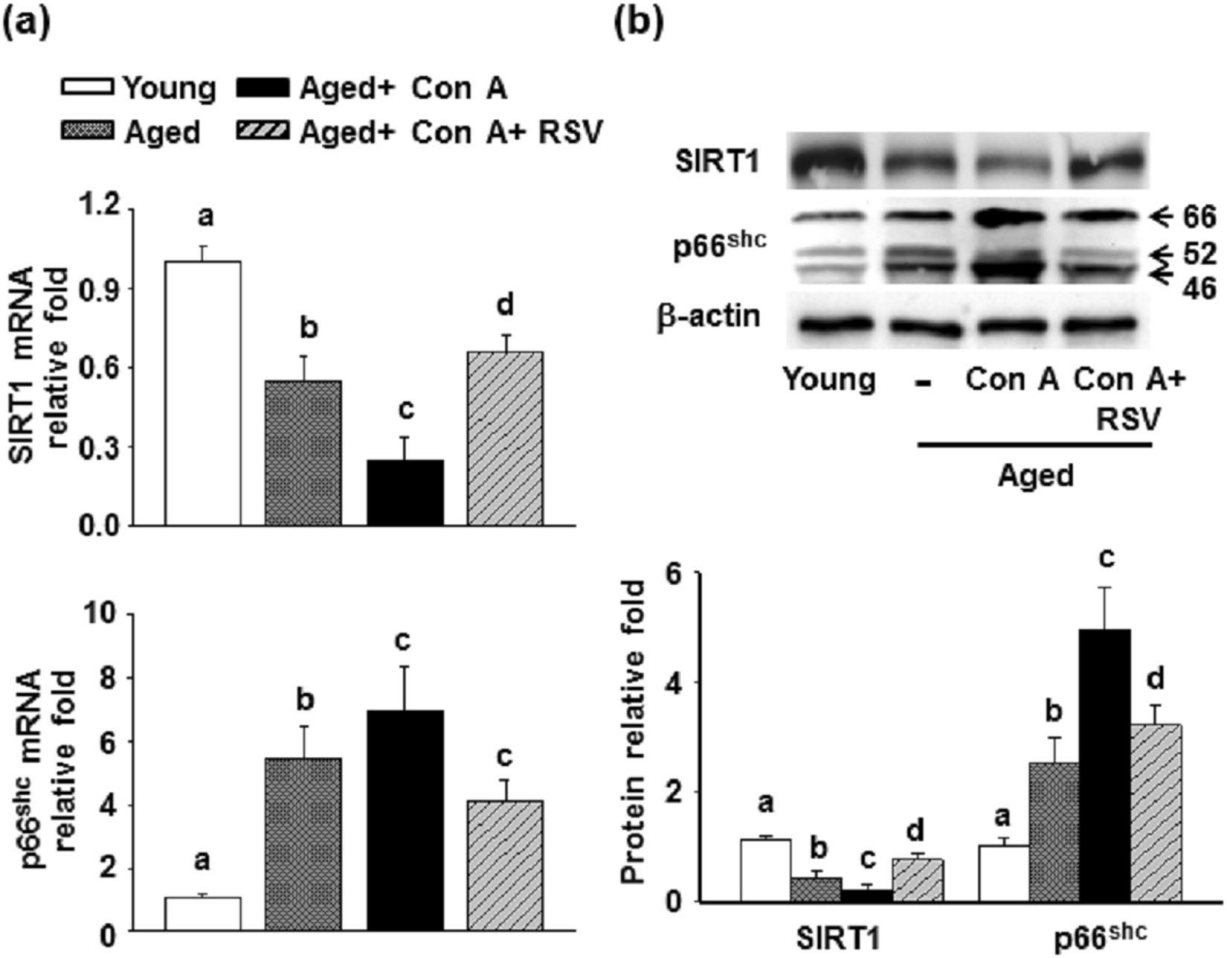
Effects of resveratrol on hepatic levels of SIRT1 and p66shc in aged mice treated with Con A. (**a**) mRNA and (**b**) protein levels of SIRT1 and p66shc in the liver tissues of mice were detected by RT-qPCR and Western blotting, respectively. The data are expressed as the means ± SEMs from three independent measurements. Different letters indicate statistically significant differences (p < 0.05) between the groups.

SIRT1 and p66^shc^ have important roles as regulators in age-related diseases^17, 21^. Our data showed that hepatic mRNA level of SIRT1 was significantly decreased, but the p66shc mRNA expression was markedly enhanced in aged mice compared with young mice (Supplementary Fig. 3A). A further reduction was observed in SIRT1 mRNA level in aged mice after Con A treatment compared to aged mice. Western blotting data showed that the protein level of SIRT1 was reduced, and p66shc protein was augmented significantly in aged mice in comparison with young mice (Supplementary Fig. 3B). Furthermore, the p66shc protein expression was presented about 5- and 1.6-fold enhancement in liver from aged mice after Con A treatment compared to young mice and aged mice, respectively. Conversely, the protein level of SIRT1 was lower in aged mice which were administrated with Con A than aged mice (Supplementary Fig. 3B). Afterwards, we explored whether alterations of SIRT1 and p66^shc^ levels were associated with the hepatoprotective effects of resveratrol on Con A-induced liver injury in aged mice. Hepatic mRNA level of SIRT1 significantly decreased while p66^shc^ mRNA level increased in the aged mice compared with the young mice. Further changes in these phenomena were observed in aged mice after Con A treatment (Fig. 3a). Similar changes occurred in the protein expression of SIRT1 and p66^shc^ in the liver of the mice (Fig. 3b). In contrast, pretreatment with resveratrol reversed Con A-mediated elevation of p66^shc^ and reduction of SIRT1 level in the livers of aged mice.

### Effects of resveratrol on Con A-induced oxidative stress and apoptotic cell death in livers of aged mice

**Figure 4:**
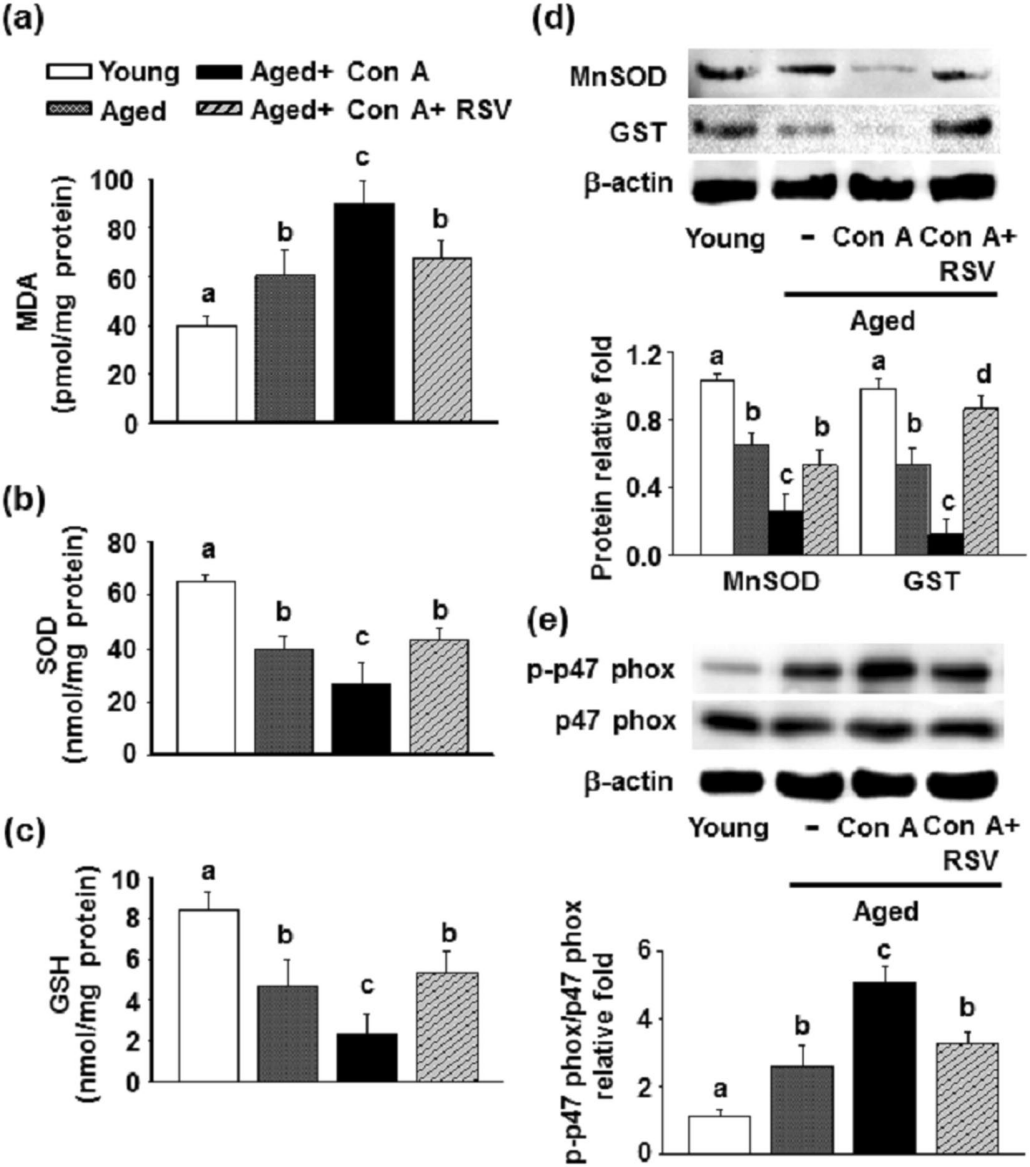
Effects of resveratrol on oxidative stress in the livers of aged mice challenged with Con A. (**a**) Hepatic lipid peroxidation levels represented as MDA equivalents (n = 7 in each group). (**b**) Hepatic SOD and (**c**) GSH assays (n = 7 in each group). (**d**) The protein levels of the MnSOD, GST, and (**e**) total and phosphorylated forms of p47 phox were determined by Western blotting. The results are expressed as the means ± SEMs from three independent measurements. Different letters denote statistically significant differences (p < 0.05) between groups.

Numerous reports have revealed potential therapeutic approaches to improve Con A-induced liver injury through suppression of oxidative stress and apoptotic cell death^34–36^. Our results indicated that the MDA level was significantly increased in the aged mice compared with that of the young mice, and a further elevation was observed in aged mice after Con A challenge (Fig. 4a). Additionally, aged mice had significantly less hepatic superoxide dismutase (SOD) and glutathione (GSH) activities compared to the young mice. Con A challenge exacerbated the aged-mediated reduction of hepatic of SOD and GSH activity in the mice (Fig. 4b and c). A similar situation occurred with hepatic protein levels of MnSOD and glutathione *S*-transferase (GST) (Fig. 4d). Pretreatment with resveratrol significantly reduced the increase in the MDA level and ameliorated the decreases of SOD and GSH activity as well as the protein level of MnSOD and GST in the liver of aged mice with Con A challenge. Additionally, p47 phox, known as a subunit of the NADPH oxidase complex conducts reactive oxygen species (ROS) in response to neutrophil stimulation^37^. Our result revealed that p47 phox phosphorylation was increased in aged mice compared with young mice, and this incremental phosphorylation was more vigorous in aged mice after a challenge with Con A (Fig. 4e). Resveratrol administration significantly suppressed the Con A-induced increase in p47 phox phosphorylation in aged mice.

**Figure 5:**
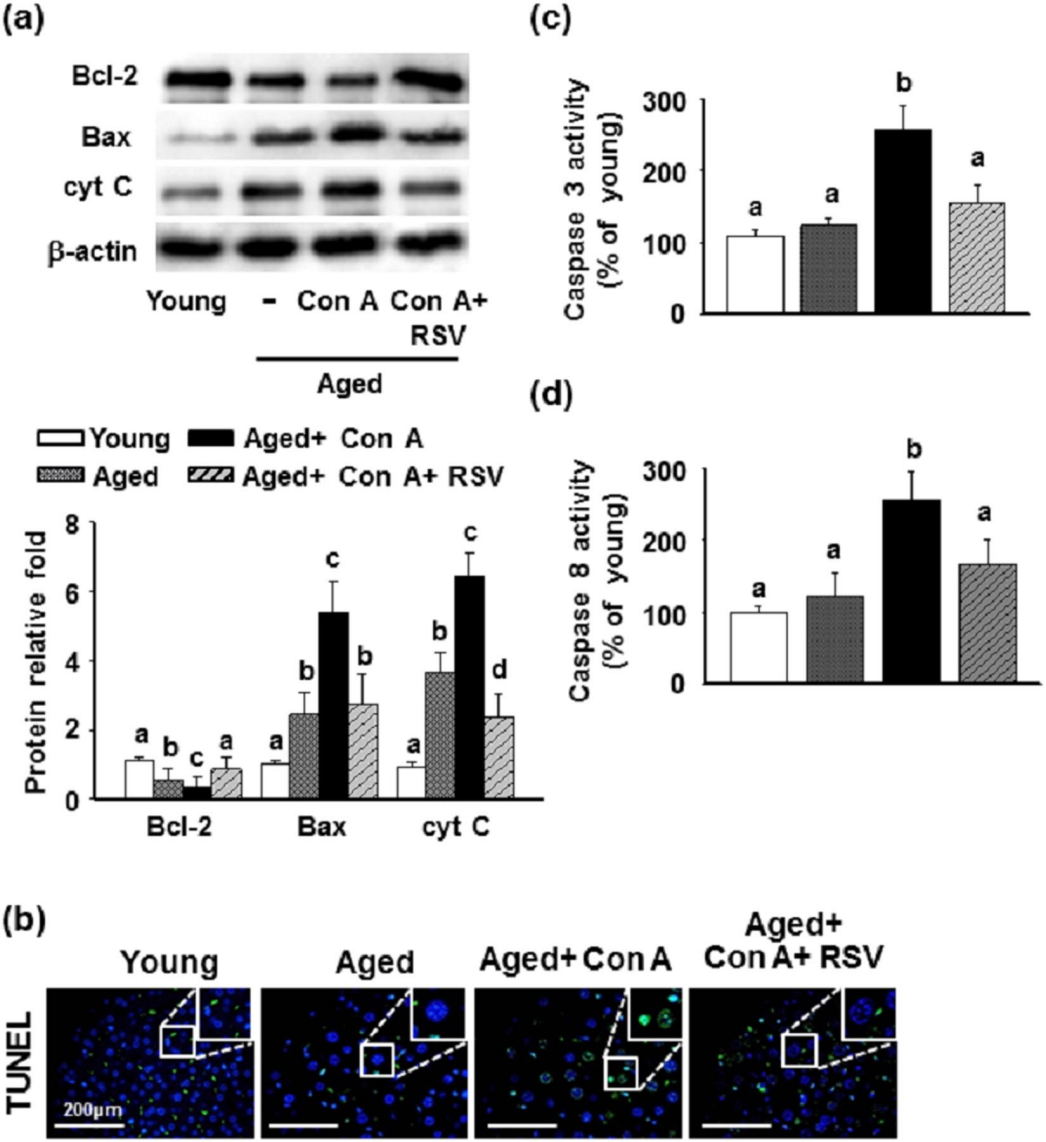
Effects of resveratrol on apoptotic cell death in the livers of aged mice treated with Con A. (**a**) Protein lysates were analyzed using Western blotting with the antibodies against Bcl-2, Bax and cytochrome C (cyt C). Data are means ± SEMs from three independent measurements. (**b**) TUNEL staining with FITC fluorescein label. DAPI is the nuclear stain. The images are shown at 200 × magnification, with the upper right grid at 400 × enlargement for selected squares. The length of scale bar was indicated as 200 μm. (**c** and **d**) The hepatic activity of caspase 3 and caspase 8 (n = 7 in each group). Different letters denote statistically significant differences (p < 0.05) between groups.

A previous study indicated that intracellular ROS arise before cytochrome C release during the activation of several apoptotic pathways^38^. Thus, we further explored whether resveratrol attenuated apoptotic cell death in the livers of aged mice after treatment with Con A. As shown in Fig. 5a, the hepatic levels of Bax and cytochrome C were increased, but Bcl-2 expression was reduced significantly in aged mice compared to young mice. After a Con A challenge, the aged mice underwent further increases in Bax and cytochrome C levels while Bcl-2 expression was inhibited in the liver. Moreover, the hepatocytes began to develop the characteristic morphology of apoptotic cells, with large round nuclei (Fig. 5b, blue fluorescence site in the upper right grid). The cells underwent chromatin condensation and cleavage of the genomic DNA in Con A-treated aged mice (Fig. 5b, green fluorescence sites). However, these effects were reversed by pretreatment with resveratrol.

The caspase family of cysteine proteases plays key roles in both the initiation and execution of apoptosis, although no statistically significant differences were observed in the hepatic activities of caspase 3 or caspase 8 between the young and aged mice. However, the activities of the two caspases were increased in aged mice challenged with Con A, and these phenomena were suppressed by pretreatment with resveratrol (Fig. 5c and d).

### Effect of resveratrol on Con A-induced impairment of liver regeneration in aged mice

**Figure 6:**
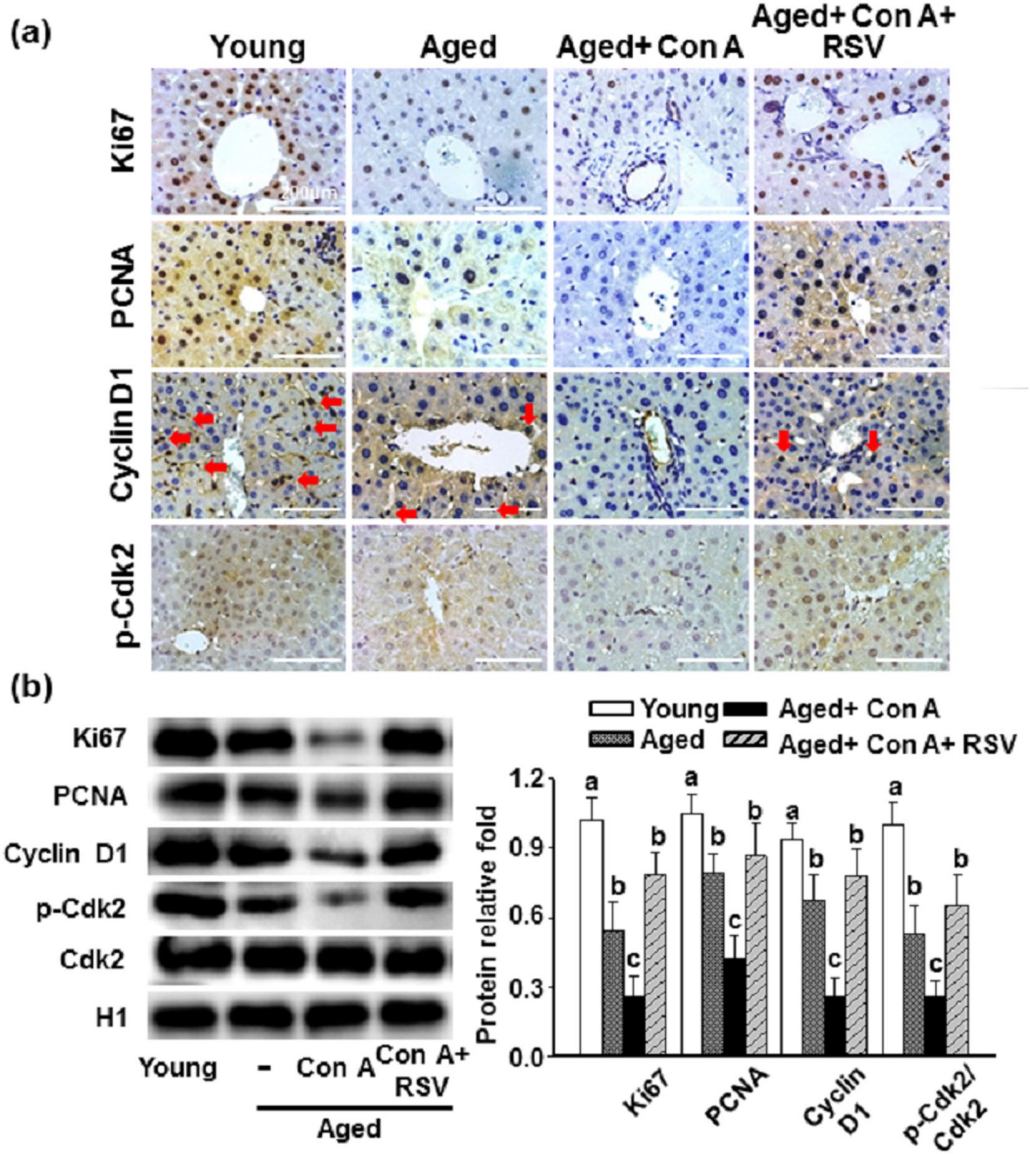
Effects of resveratrol on impairment of liver regeneration in aged mice treated with Con A. (**a**) Representative IHC stain for Ki67, PCNA, cyclin D1, and phosphorylated Cdk2; dark brown color, red arrows depicted sites of cyclin D1. The results are shown at 200 × magnification and the length of scale bar was indicated as 200 μm. (**b**) Hepatic protein levels of Ki67, PCNA, cyclin D1 and total and phosphorylated forms of Cdk2 were determined by Western blotting. The results are expressed as the means ± SEMs from three independent measurements. Different letters indicate statistically significant differences (p < 0.05) between groups.

It is reported that T cell–mediated hepatitis induced by Con A inhibits the normal regenerative response of the liver^13^. As illustrated in Fig. 6a, aged mice showed reduced Ki67, proliferating cell nuclear antigen (PCNA), cyclin D1, and p-cyclin-dependent kinase 2 (Cdk2) levels in the liver compared with young mice. Further reductions were observed after treatment of aged mice with Con A. Western blot results showed a similar pattern (Fig. 6b). In contrast, resveratrol reversed the Con A-inhibition of Ki67, PCNA, cyclin D1 and p-Cdk2 expressions in the livers of aged mice.

## Discussion

The prevalence of chronic liver disease is increasing in the elderly population. Liver regeneration from periportal progenitor cells to mature hepatocytes is impaired in autoimmune liver failure, resulting in resistance to immunosuppressive therapy^39^. It is reported that resveratrol ameliorates experimental autoimmune myocarditis and inflammatory arthritis in animal models^40, 41^. The present study demonstrated that Con A-aggravated immune dysregulation of livers in aged mice further contributed to piecemeal necrosis, oxidative stress, apoptotic cell death, and impairment of liver regeneration. This immune dysregulation occurred through downregulation of SIRT1 expression and upregulation of p66^shc^. Pretreatment with reveratrol reversed these phenomena. Our findings also confirmed a correlation between SIRT1 and p66^shc^ expression in the mouse model.

Immune aging, also known as immunosenescence, is defined as an age-related decline in populations and functions of the immune system, which is partially responsible for the increased prevalence and severity of infectious diseases because a persistent inflammatory response is involved in this process^2, 42^. The histopathological characteristics of the livers showed that the sizes of neutrophil and T lymphocyte populations mildly increased in aged mice compared to the young mice (Fig. 1). However, plasma levels of inflammatory cytokines in the aged mice were higher than in the young mice. Moreover, a dramatic elevation of immune responses and inflammation occurred in the aged mice after exposure to Con A. It is noteworthy that thymic atrophy in advancing age contributes to the progressive reduction of the naïve T cell fraction and the relative increase of the memory T cell subset^43^. Speziali *et al*. revealed that aged mice presented with greater proliferation of spleen T cells than in young mice after exposure to *in vitro* stimulation with Con A because senescence is associated with an increase in T cell activation represented by the activity of memory cells^44^. The situation is similar for age-associated CD8 T cell activation^45^. Moreover, the aging process is associated with an increase in the pro-inflammatory status of the organism^42^. The NF-κB is a transcription factor that promotes the expression of pro-inflammatory genes that activate immune responses^46^. The roles of iNOs, PAI-1, and MCP-1 are involved in Con A-mediated hepatitis^47–49^. Therefore, the expressions of these inflammatory cytokines in our studies demonstrated that the condition of aging is more susceptible to immunosenescence-mediated inflammatory injury in livers challenged with Con A.

There are seven sirtuin homologs in mammals, and SIRT1 has received the most attention among them. The regulatory role of SIRT1 in cell proliferation and apoptosis was predicted based on the activation of the NF-κB pathway^50^ in lymphocytes. SIRT1 negatively regulates the transcriptional activity of NF-κB p65 to modulate the immune response in macrophages^51^. In agreement with a previous report, our results showed that aged mice challenged with Con A had higher levels of NF-κB p65, macrophage accumulation, and a reduction of SIRT1 expression in their livers compared with that of untreated aged mice. Nonetheless, these characteristics were reversed by pretreatment with resveratrol. The p66^shc^ protein acts as a regulator of cellular responses to oxidative stress, apoptosis, and aging^19, 20^, and its expression rises in the elderly^52^. A plethora of evidence indicates that p66^shc^-mediated oxidative stress is a conceivable key factor in upstream apoptosis^38, 53, 54^. Additionally, SIRT1 negatively regulates p66^shc^ ^24–26^.

Con A-induced hepatotoxicity triggers broad apoptosis and necrosis leading to the death of hepatocytes^55^. Apoptosis is initiated through an extrinsic pathway that is triggered by engagement of the death initiation signaling complex-caspase 8 cascade, a death receptor, or an intrinsic pathway that is induced by mitochondrial injury (cytochrome C release) and influenced by members of the Bcl-2 family. Thus, we hypothesize that hepatic oxidative stress and apoptotic cell death are involved in the severe impairment of the immune system in the livers of aged mice when exposed to Con A. Our findings revealed that hepatic p66^shc^ expression was significantly greater in aged mice compared with young mice. Moreover, Con A treatment led to the aggravation of p66^shc^ expression, particularly in aged mice. Correspondingly, a parallel augmentation occurred in (1) hepatic oxidative stress, including lipid peroxide production (indicated as the MDA level) and p47 phox phosphorylation, and (2) apoptotic cell death, including expression of Bax and cytochrome C, caspase 3 and caspase 8 activity, and the appearance of terminal deoxynucleotidyl transferase-mediated dUTP nick end labeling (TUNEL)-labeled cells in the livers of aged mice challenged with Con A. Conversely, the antioxidant activities of SOD and GSH and protein levels of MnSOD and GST as well as the anti-apoptotic oncoprotein Bcl-2 were reduced by Con A challenge in aged mouse livers. The initiation of the intrinsic pathway of apoptosis resulted from intramitochondrial cytochrome C release into the cytosol. Notably, p66^shc^ is a redox enzyme that generates mitochondrial hydrogen peroxide (H_2_O_2_) as a signaling molecule for apoptosis by oxidizing cytochrome C and reducing equivalents of the mitochondrial electron transfer chain^38^. MnSOD is the primary mitochondrial ROS scavenging enzyme that converts superoxide to H_2_O_2_, and the GSH redox cycle is a major endogenous antioxidant that provides cellular protection against H_2_O_2_ and certain organic hydroperoxides. Therefore, in keeping with our general scheme, we suggest that an enhanced level of p66^shc^, induced by a Con A challenge in aged mice, is positively associated with upregulation of hepatic oxidative stress and apoptotic cell death. We also illustrated that resveratrol suppressed Con A-induced increases in hepatic p66^shc^ levels by upregulation of SIRT-1 expression. Importantly, Con A-induced liver injury is driven by aggravation of hepatic oxidative stress and hepatocyte apoptosis in aged mice. Resveratrol attenuated the hepatic oxidative stress.

Liver regeneration is a multistep process that may be divided into priming pathways, growth-promoting pathways, and growth-inhibitory pathways. It is reported that compared with young mice, old mice showed a significantly lower expression of hepatocyte growth factor, cMet, cyclin D1, cyclin A2, and PCNA as well as a markedly elevated level of caspase-3 after partial hepatectomy^10^. In addition, Zhang and colleagues indicate that intravenous injection of IL-22 could enhance liver regeneration in mice with Con A-induced hepatitis after partial hepatectomy by augmentation of PCNA and cyclin D1 levels^14^. Our results revealed that pretreatment with resveratrol promoted hepatic levels of Ki67, PCNA, cyclin D1 and Cdk2 in aged mice challenged with Con A, and these findings are similar to those of Zhang *et al*.^14^. However, the limitations of our study is that no long-term confirmation of the protective effects of resveratrol on the neutralization of Con A-aggravated immune dysregulation and dysfunction of liver regeneration in aged livers was performed using knockout mice deficient in SIRT1.

In conclusion, a better understanding of the mechanisms underlying age-related liver changes may help to preserve hepatic function and improve morbidity and mortality. Resveratrol pretreatment conferred protection against Con A-induced aggravation of hepatitis in aged mice by attenuating aberrations of immune response and liver regeneration, at least in part, through SIRT1-mediated repression of p66^shc^ expression. This concept requires extensive research and further clinical proof of efficacy, followed by translational studies in the future.

## Materials and Methods

### Animals and experimental protocols

Male C57BL/6 mice were purchased from the National Laboratory Animal Center (Taipei, Taiwan) and housed in an air-conditioned room at 20 ± 2 °C with a 12-hour light/dark cycle. Mice were divided into four groups: (1) untreated young mice (4 to 6 months of age) (n = 7), (2) untreated aged mice (24 months of age) (n = 7), (3) Con A-treated aged mice (n = 7 or 8), (4) resveratrol + Con A-treated aged mice (n = 7 or 8). Mice were orally administered the vehicle [0.5% (w/v) sodium carboxymethyl cellulose] or 30 mg/kg body weight of resveratrol (Sigma-Aldrich, St. Louis, MO) seven times at an interval of 12 h. Twelve hours after the last oral resveratrol dose, Con A (Sigma-Aldrich) was given to the 3^rd^ and 4^th^ groups of mice at a dose of 20 mg/kg by infusion into the tail vein for 8 h to induce liver injury. The control mice (groups 1 and 2) were treated with an equal volume of pyrogen-free saline. The mice were euthanized by CO_2_ inhalation before decapitation. The plasma and liver tissues were obtained for further analysis. The experimental procedures were approved by the Chang Gung University Animal Care and Use Committee (IACUC Approval No.: CGU12-133) in accordance with international standards of humane animal use.

### Cytokine assays

Analyses of plasma ALT and AST were performed using a commercially available diagnostic kit (Randox Laboratories, Antrim, UK). The plasma levels of TNF-α, IL-6, IFN-γ, and MCP-1 were determined using a commercial ELISA kit (R&D system, Minneapolis, MN). Liver levels of SOD, GSH, caspase 3, and caspase 8 were measured using a superoxide dismutase assay kit and glutathione assay kit (Cayman, MI, USA) and caspase 3 and caspase 8 assay kits (Abcam, Cambridge, UK) according to the manufacturer’s instructions.

### Liver histological stain assay

Liver specimens were fixed in 4% paraformaldehyde and embedded in paraffin, followed cutting into 5-μm-thick sections, which were then stained with hematoxylin and eosin (H&E). For immunohistochemical (IHC) and immunofluorescence (IF) assays, slides were deparaffinized and rehydrated with ethanol, then sequentially incubated with 0.3% H_2_O_2_ to block endogenous peroxidase activity. Next, the slides were incubated with various primary antibodies including F4/80, neutrophil, PCNA, cyclin D1, p-Cdk2 (all from Abcam), CD4, CD8 (all from BD Bioscience, San Jose, CA), and Ki67 (Millipore, Temecula, CA) for 2 h at room temperature. Subsequently, a biotinylated secondary antibody and avidin-biotin complex reagent were added, and color development was induced by DAB and visualized under a light microscope for IHC. For immunofluorescence assays, DAPI was used as the nuclear stain and was visualized using a fluorescence microscope for CD4 and CD8.

### Western blotting assay

Frozen liver tissue (30 mg) was homogenized in 0.5 ml CelLytic M lysis reagent (Sigma-Aldrich) containing 1% phosphatase inhibitor cocktail and protease inhibitor cocktail, and centrifuged at 13,000 × *g* for 30 min at 4 °C. The protein concentration of the supernatant was determined by the Bradford assay. The cell lysates were separated using SDS-PAGE and transferred onto PVDF membranes, followed incubation with primary antibodies against NF-κB p65, MnSOD, Bax, Bcl-2 (all from Santa Cruz Biotechnology, Dallas, TX), p66^shc^, p-p47 phox, p47 phox, cytochrome C (all from Millipore), PAI-1, MCP-1, Cdk2 (all from Abcam), iNOs (BD Bioscience), SIRT1 (AVIVA Systems Biology, San Diego, CA), and GST (Cell Signaling, Danvers, MA). Finally, horseradish peroxidase-conjugated secondary antibodies were used for ECL detection. The results were normalized against β-actin and H1 as internal controls.

### RT-qPCR assay

Total RNA was extracted using the guanidinium-phenol-chloroform method. Reverse transcription was carried out with the RevertAid First Strand cDNA Synthesis kit (Thermo Scientific, Waltham, MA) according to the manufacturer’s instructions. Samples were subjected to real-time PCR on a LightCycler 1.5 apparatus, and the reaction was carried with a LightCycler FastStart DNA Master^PLUS^ SYBR Green I kit (Roche, Mannheim, Germany). The following primer sequences were used: p66shc, 5′-ACTACCCTGTGTTCCTTCTTTC-3′ (sense) and 5′-TCGGTGGATTCCTGAGATACTGT-3′ (antisense); SIRT-1, 5′-GCAACAGCATCTTGCCTGAT-3′ (sense) and 5′-GTGCTACTGGTCTCACTT-3′ (antisense). The relative expression of each targeted gene was normalized to the GAPDH threshold cycle (CT) value and quantified using the comparative threshold cycle 2^−∆∆CT^ method.

### Liver lipid peroxidation analysis

Liver lipid peroxidation assays were performed by measuring the production rate of thiobarbituric acid-reactive substances (TBARS) and were expressed as malondialdehyde (MDA) equivalents. Briefly, liver tissues were homogenized in ice-cold PBS containing 0.25 mM butylated hydroxytoluene and centrifuged at 13,000 × *g* for 20 min at 4 °C. The supernatant was collected for TBARS assay; 20 μl of sample was mixed thoroughly with 80 μl of reaction buffer (0.4% sodium dodecyl sulfate, 0.8% phosphotungstic acid, 0.18% thiobarbituric acid, and 0.05 N HCl) and heated for 30 min in a boiling water bath. After cooling, the mixture was added to 0.1 ml of n-butyl alcohol and centrifuged at 13,000 × *g* for 5 min at 4 °C. The amount of MDA was assessed by measuring the optical density at 535 nm at 37 °C, and the results were expressed as nmol/mg protein.

### Terminal deoxynucleotidyl transferase-mediated dUTP nick end labeling assay

TUNEL assay was conducted using the *In Situ* Cell Death Detection Kit (Roche Applied Science, Mannheim, Germany) according to the manufacturer’s instructions. Briefly, liver sections were deparaffinized and rehydrated, then washed with PBS buffer. They were then stained using the TUNEL reaction mix for 1 h at room temperature. The FITC-label TUNEL-positive cells were analyzed under a fluorescence microscope, and DAPI was used for nuclear staining. Image quantitation of TUNEL assay was also performed using HistoQuest software (TissueGenostics).

### Statistical analysis

Data are expressed as means ± SEMs and are compared using Student’s *t*-tests. P < 0.05 is considered to indicate a statistically significant difference. Comparisons among groups were made using one-way analysis of variance (ANOVA) and subsequent Tukey’s post hoc tests for multiple comparisons.

## Electronic supplementary material

Supplementary information
