# Supplementary material for: Resveratrol Pretreatment Attenuates Concanavalin A-induced Hepatitis through Reverse of Aberration in the Immune Response and Regenerative Capacity in Aged Mice
Source: Sci Rep. 2017 Jun 2;7:2705. doi: 10.1038/s41598-017-02881-z (PMC5457448; doi:10.1038/s41598-017-02881-z)
Supplement: Supplementary file 1 — Supplementary information [file 41598_2017_2881_MOESM1_ESM.pdf]

Supplementary information

Resveratrol Pretreatment Attenuates Concanavalin A-induced Hepatitis through Reverse of Aberration in the Immune Response and Regenerative Capacity in Aged Mice

Tse-Hung Huang, Chin-Chang Chen, Hsuan-Miao Liu, Tzung-Yan Lee, and Sue-Heui Shieh

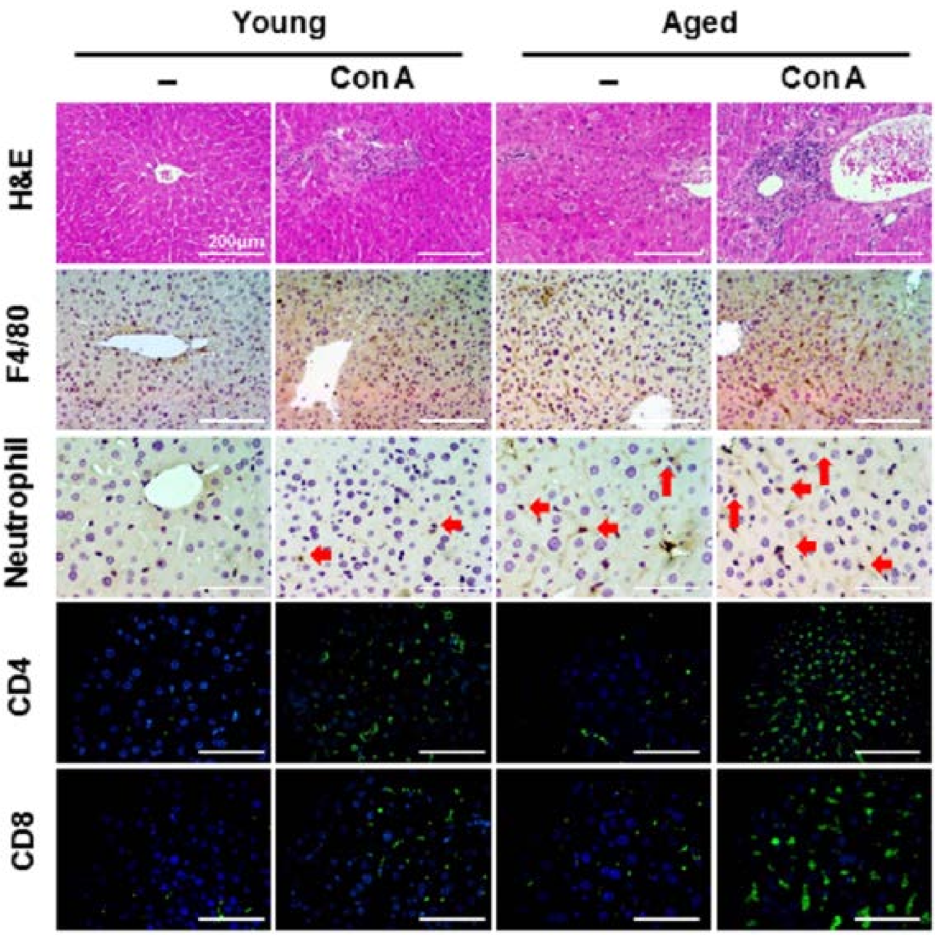

Supplementary Figure 1. Hepatic injury characteristics of young and aged mice exposed to Con A. Paraffin-embedded liver sections taken after each sacrifice were stained with H&E. Infiltration of macrophage and neutrophil in liver was stained with antibodies for F4/80 and Neutrophil, respectively. The indication was displayed as a dark brown color in the IHC stain (the red arrows depicted the sites of neutrophil). The T helper and T cytotoxic lymphocytes were stained with CD4 and CD8 antibodies, respectively. DAPI is the nuclear stain. All results were shown at 200X magnification and the length of scale bar was indicated as 200 μm.

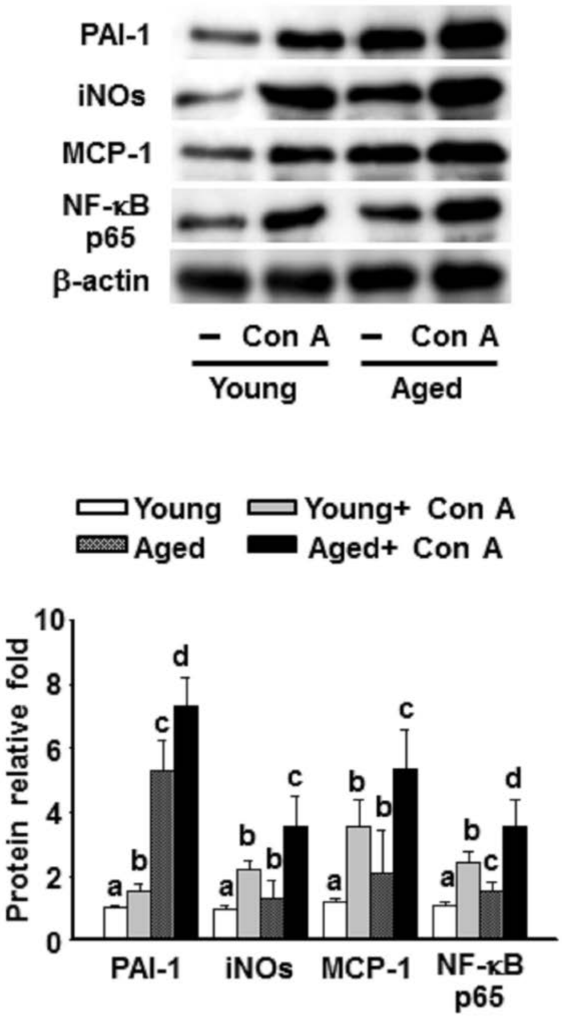

Supplementary Figure 2. Comparison of hepatic inflammatory response in young and aged mice treated with Con A. Hepatic protein levels of PAI-1, iNOs, MCP-1 and NF-κB p65 were detected using Western blotting. The results were quantified after densitometry and β-actin was used as an internal control. The data were represented as the mean ± SEM for three independent measurements. Values are significantly different letters denote statistically significant differences ($p<0.05$) between groups, according to Tukey's post hoc tests.

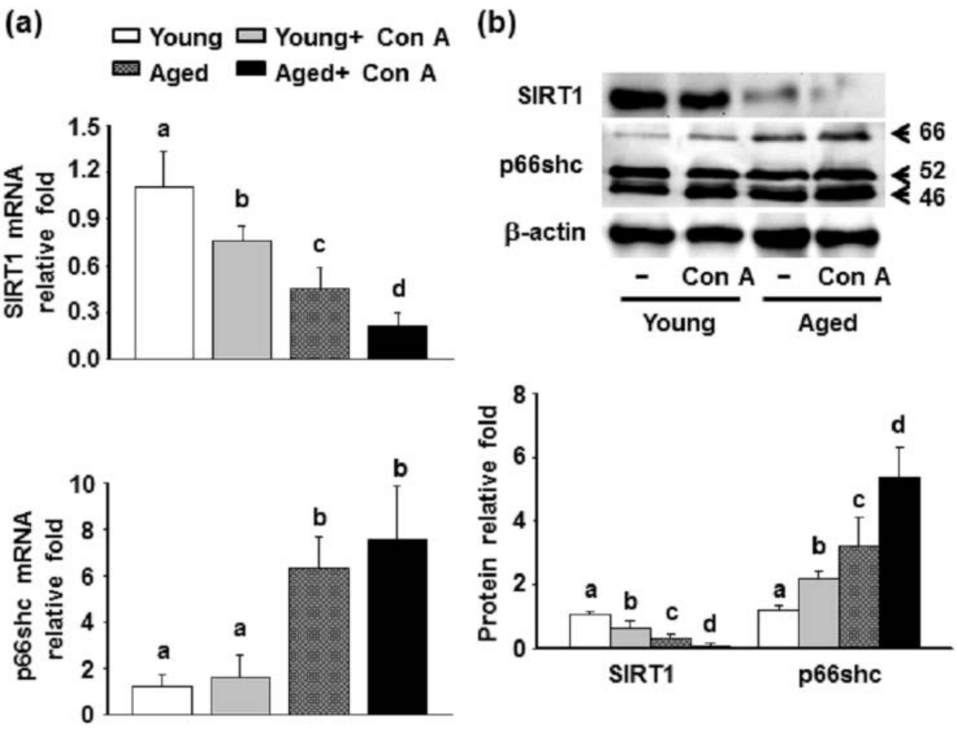

Supplementary Figure 3. Hepatic levels of SIRT1 and p66shc and SIRT1 in young and aged mice after Con A challenge. (a) mRNA and (b) protein levels of SIRT1 and p66shc in the liver from mice treatment with or without Con A were detected using RT-qPCR and Western blotting, separately. The data were represented as the mean ± SEM for three independent measurements. Different letters indicate statistically significant differences ($p < 0.05$) between the groups.
